# Molecular profiles and tumor mutational burden analysis in Chinese patients with gynecologic cancers

**DOI:** 10.1038/s41598-018-25583-6

**Published:** 2018-06-12

**Authors:** Min Wang, Wensheng Fan, Mingxia Ye, Chen Tian, Lili Zhao, Jianfei Wang, Wenbo Han, Wen Yang, Chenglei Gu, Mingxia Li, Zhe Zhang, Yongjun Wang, Henghui Zhang, Yuanguang Meng

**Affiliations:** 1 Department of Gynecology and Obstetrics, Chinese PLA General Hospital, Beijing, P.R. China; 2 Department of Gynecology and Obstetrics, The 306th Hospital of PLA, Beijing, P.R. China; 3 Institute of Infectious Diseases, Beijing Ditan Hospital, Capital Medical University, Beijing, P.R. China; 4 Beijing Genecast Biotechnology Co., Beijing, P.R. China; 5 Department of Gynecology and Obstetrics, Peking University International Hospital, Beijing, P.R. China; 6 Department of Gynecology and Obstetrics, Beijing Obstetrics and Gynecology Hospital, Capital Medical University, Beijing, P.R. China

## Abstract

The goal of this work was to investigate the tumor mutational burden (TMB) in Chinese patients with gynecologic cancer. In total, 117 patients with gynecologic cancers were included in this study. Both tumor DNA and paired blood cell genomic DNA were isolated from formalin-fixed paraffin-embedded (FFPE) specimens and blood samples, and next-generation sequencing was performed to identify somatic mutations. *TP53*, *PTEN*, *ARID1A*, and *PIK3CA* alterations were significantly different in various types of gynecologic cancers (p = 0.001, 1.15E-07, 0.004, and 0.009, respectively). The median TMB of all 117 gynecologic tumor specimens was 0.37 mutations/Mb, with a range of 0–41.45 mutations/Mb. Despite the lack of significant difference, endometrial cancer cases had a higher median TMB than cervical and ovarian cancer cases. Younger gynecologic cancer patients (age <40 years) had a significantly lower TMB than older patients (age ≥40 years) (p = 0.04). In addition, TMB was significantly increased with increasing clinical stage of disease (p = 0.001). *PTEN* alterations were commonly observed in patients with a moderate to high TMB (n = 8, 38.10%, p = 9.95E-04). Although limited by sample size, all of the patients with *TSC2* (n = 3, p = 3.83E-11) or *POLE* (n = 2, p = 0.005) mutations had a moderate to high TMB. Further large-scale, prospective studies are needed to validate our findings.

## Introduction

Gynecologic oncology is a specialized field of medicine that focuses on cancers of the female reproductive system, including cancer of the cervix, ovaries, uterus, fallopian tubes, vagina and vulva. Gynecologic cancers annually affect approximately 100 million people worldwide and account for approximately 18% of all female cancers^1^. Cervical cancer is the most common cancer of the female reproductive system in China^2^. According to the latest data^3^, 100 thousand new cases of cervical cancer occurred in 2013. Additionally, an estimated 98.9 thousand new cervical cancer cases and an estimated 30.5 thousand associated deaths were predicted in 2015^4^. The overall five-year relative survival rate for cervical cancer is 45.4% in China^5^. Endometrial cancer is the second-most common cancer of the female reproductive system in China^2^. In 2013, 61.9 thousand new cases of endometrial cancer were reported. Furthermore, endometrial cancer was estimated to account for 63.4 thousand new cancer cases in 2015^4^. Moreover, the number of disease-specific deaths was estimated to increase from 17.9 thousand in 2013 to 21.8 thousand in 2015^3,4^. The overall five-year relative survival rate for endometrial cancer is 55.1% in China^5^. Ovarian cancer is the third-most common cancer of the female reproductive system in China^2^. Approximately 50 thousand new ovarian cancer cases occurred in 2013, while 52.1 thousand new cases were estimated to have occurred in 2015^3,4^. In 2013, 21.3 thousand patients died of ovarian cancer^5^. Ovarian cancer was estimated to account for 22.5 thousand deaths in 2015^4^. The overall five-year relative survival rate for ovarian cancer is 38.9% in China^5^. The survival rate for ovarian cancer is lower than that for cervical and endometrial cancer. The major reason for this poor survival is that ovarian cancer is commonly diagnosed at an advanced stage due to its anatomic location. Survival rates decrease sharply with increasing stage of the disease. The five-year relative survival rates of ovarian cancer were 90.2%, 68.3%, 32.9%, and 16.1% for stage I, II, III, and IV, respectively, according to data from a historical cohort study in Hong Kong^6^; however, the data at the national level are still not available. Traditionally, the management of women with gynecologic cancer is predominantly based on surgery, cytotoxic chemotherapy and radiotherapy, either alone or in combination, as dictated by the clinical circumstances, with the stage of disease largely determining the need for adjuvant or first-line chemotherapy or radiation. However, approximately 70% of patients experience disease relapse after varying disease-free intervals. PEGylated liposomal doxorubicin, topotecan, and gemcitabine are among the cytotoxic agents used in the platinum-resistant setting, with generally low response rates (RRs)^7–9^. More recently, an increasing number of targeted therapies directed against a variety of molecular targets in gynecologic cancers and their microenvironments have been developed and used in women with these malignancies^10^. Among these targeted therapies, increasing evidence supports a potential role for immune checkpoint inhibitors as a viable therapeutic strategy in gynecologic cancers^11–17^. Although encouraging results have been reported in early clinical trials, immune checkpoint inhibitors exhibit limited response in cancers, and none of the new drugs is being studied in prospective phase III trials^16,18,19^. Given the potential that these agents have shown for the treatment of refractory disease and durable responses in some cases, there is great interest in identifying patients who are most likely to benefit from these therapies. In addition to PD-1/PD-L1 expression, there are other factors that can affect responses to immunotherapy, including mismatch repair deficiency, microsatellite instability (MSI), *POLE* mutations and tumor mutational burden (TMB)^15,20–24^. In fact, in one clinical trial, TMB was more significantly associated with RR than was the expression of PD-L1 by immunohistochemistry^25^. Neoantigen load has also been correlated with response to immunotherapy^26^.

To date, no study has reported on TMB in Chinese populations with gynecologic cancer. Here, we used a designed 1.15-megabase (Mb) panel to analyze the range and frequency of hypermutations among and within different types of gynecologic cancers. Furthermore, we characterized and identified specific genes associated with an increased TMB in Chinese patients with gynecologic cancer.

## Results

### Patient characteristics

In this study, the patient age ranged from 16 to 80 years with a median of 55 years (Table 1). Most tumors originated in the ovary: 68 cases were ovarian cancer including 1 case of primary peritoneal cancer and 3 cases of fallopian tube cancer; 32 cases were cervical cancer; and 17 cases were endometrial cancer. Of the 117 patients, 17.95% had stage I, 29.06% had stage II, 36.75% had stage III, and 12.82% had stage IV disease, and we were unable to determine the stage for 4 cases. According to the 2014 WHO Classification of Tumors of the Female Reproductive Organs^27^, 60 (51.28%) cases were serous adenocarcinoma including 2 cases with minor clear cell adenocarcinoma components, of which 59 cases occurred in the ovary and 1 case was endometrial; 3 (2.56%) cases were clear cell adenocarcinoma in the ovary; 17 (14.53%) cases were endometrioid adenocarcinoma, of which one case occurred in the ovary whereas the remainder were endometrial; 31 (26.5%) cases were squamous cell carcinoma in the cervix; 1 (0.85%) case was mucinous adenocarcinoma in the cervix; and 5 (4.27%) cases were of other histologic types in the ovary, including one case with osteosarcoma, one case with immature teratoma, one case with sex cord-mesenchymal tumor, and two cases with dysgerminoma. For cervical cancer, HPV status was checked in 14 patients. Nine patients were HPV positive, whereas five patients were HPV negative. Among the patients with endometrial carcinoma included in this study, most (16/17, 94.12%) were classified as type I, including one case with squamous differentiation. One patient with serous adenocarcinoma was identified as type II endometrial carcinoma.

**Table 1:** Characteristics of studied group.

	Numbers of patients (n = 117)
Age, median (range), years	55 (16–80)
<40	14 (11.97%)
40–49	26 (22.22%)
50–59	43 (36.75%)
>=60	34 (29.06%)
Site of Disease	
Ovary (include primary peritoneal and fallopian tube)	68 (58.12%)
Cervix	32 (27.35%)
Endometrial	17 (14.53%)
Stage of Disease	
stage I	21 (17.95%)
stage II	34 (29.06%)
stage III	43 (36.75%)
stage IV	15 (12.82%)
NA	4 (3.42%)
HPV status (Cervix cancer)	
positive	9 (28.13%)
negative	5 (15.63%)
uncertain	18 (56.25%)
Subtype of Endometrial Carcinoma	
Type I	16 (94.12%)
Type II	1 (5.88%)
Tissue types (in each cancer types)	
Ovarian cancer	
Serous adenocarcinoma	59 (86.76%)
Clear cell adenocarcinoma	3 (4.41%)
Endometroid adenocarcinoma	1 (1.47%)
Other	5 (7.35%)
Cervical cancer	
Squamous cell carcinoma	31 (96.88%)
Mucinous adenocarcinoma	1 (3.12%)
Endometrial cancer	
Endometroid adenocarcinoma	16 (94.12%)
Serous adenocarcinoma	1 (5.88%)

### Genomic profiles of gynecologic cancers

**Figure 1:**
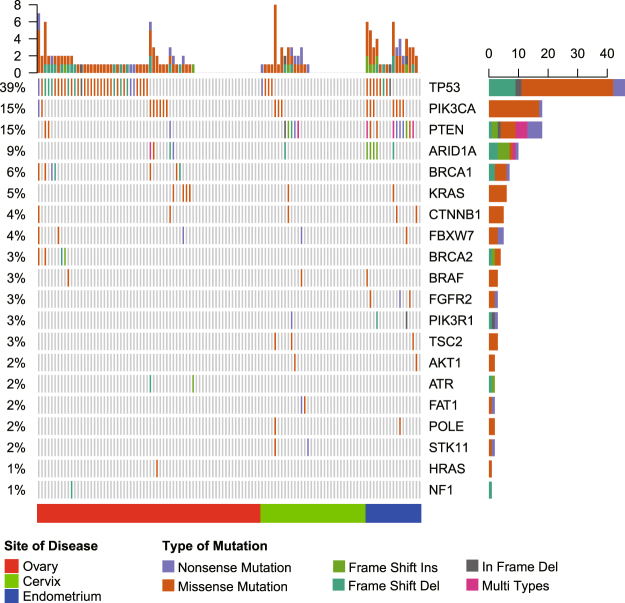
Heat map showing somatic mutation profiles of gynecologic cancers.

The genomic profiles of the 117 gynecologic cancer specimens were analyzed by using a designed panel which covered 1086 cancer-related genes (Supplemental Table S1). Out of the 117 cases, genomic alterations were identified in 79 (67.52%) including 48 (70.59%) ovarian cancer cases, 15 (46.88%) cervical cancer cases, and 16 (94.12%) endometrial cancer cases. The genomic profiles of the three types of cancers were distinct. In the cases with ovarian cancer (including primary peritoneal cancer and fallopian tube cancer), the most frequently altered genes were *TP53* (50%), *PIK3CA* (12%), *BRCA1* (10%), *ARID1A* (6%), and *BRCA2* (6%) (Fig. 1). In the cases with cervical cancer, the most frequently altered genes were *PTEN* (16%), *TP53* (12%), *PIK3CA* (9%), *FAT1* (6%), *STK11* (6%), and *TSC2* (6%) (Fig. 1). Finally, in the cases with endometrial cancer, the most frequently altered genes were *PTEN* (59%), *TP53* (47%), *PIK3CA* (41%), *ARID1A* (29%), and *FGFR2* (18%) (Fig. 1). *TP53* alterations were significantly more common in ovarian and endometrial cancer cases than in cervical cancer cases (50% and 47% vs 12%, p = 0.001). Additionally, *ARID1A*, *PIK3CA* and *PTEN* alterations were significantly more common in endometrial cancer cases than in ovarian and cervical cancer cases (*ARID1A*: 29% vs 6% and 3%, p = 0.004; *PIK3CA*: 41% vs 12% and 9%, p = 0.009; *PTEN*: 59% vs 4% and 16%, p = 1.15E-07), whereas *BRCA1* and *BRCA2* somatic mutations were found only in patients with ovarian cancer.

### TMB of gynecologic cancers

**Figure 2:**
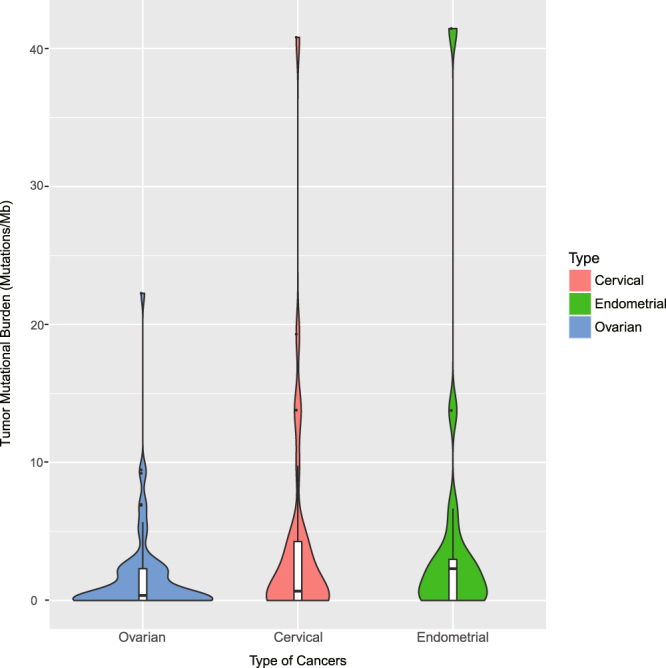
TMB levels of gynecologic cancers.

We next investigated the TMB of gynecologic cancers and their association with clinical status. Across the entire dataset, the median TMB was 0.37 mutations/Mb, with a range of 0–41.45 mutations/Mb. Out of the 117 patients, 96 (82.05%) had a low TMB, 14 (11.97%) had a moderate TMB, and 7 (5.98%) had a high TMB. Although the TMB of different age groups was not significantly different (p = 0.37), younger patients with gynecologic cancer (age <40 years) had a significantly lower TMB than older patients (age ≥40 years) (Table 2 and Supplementary Table S2, p = 0.04). In addition to age, different cancer types also showed various TMB distribution patterns. High TMB values were found in all gynecologic cancer types – 8 (11.76%) ovarian cancer cases had a moderate to high TMB value including one case with a high TMB; 4 (23.53%) endometrial cancer cases had a moderate to high TMB including 2 cases with a high TMB; and 9 (28.13%) cervical cancer cases had a moderate to high TMB including 4 cases with a high TMB. Despite the lack of statistically significant difference, patients with endometrial cancer had a higher median TMB than patients with cervical and ovarian cancer (including primary peritoneal and fallopian tube cancer) (2.30 vs 0.67 and 0.36 Fig. 2 and Table 2), indicating that immune checkpoint blockade may be a potential treatment for endometrial cancer patients. In cervical cancer, TMB did not differ significantly according to HPV status (Table 2). Additionally, TMB was significantly associated with the clinical stage of gynecologic cancer (Table 2, p = 0.001). Increasing stage of tumors was associated with increasing median TMB – 0 mutations/Mb for stage I, 0 mutations/Mb for stage II, 0.83 mutations/Mb for stage III, and 2.92 mutations/Mb for stage IV disease. Although endometrioid adenocarcinoma cases had a relatively increased median TMB, there was no statistically significant difference in TMB values among gynecologic cancers with different histologies (Table 2 and Supplementary Table S2, p = 0.34).

**Table 2:** The association of TMB levels and clinical status.

		TMB, median (range), mutations/Mb	P value^a^	TMB level		P value^b^
				Low (%)	Moderate to high (%)	
Age, median (range), years			0.04			0.26
<40	14	0 (0–4.80)		13 (92.86%)	1 (7.14%)	
>=40	103	0.67 (0–41.45)		83 (80.58%)	20 (19.42%)	
Site of Disease			0.81			0.11
Ovary (include primary peritoneal and fallopian tube)	68	0.36 (0–22.26)		60 (88.24%)	8 (11.76%)	
Cervix	32	0.67 (0–40.81)		23 (71.87%)	9 (28.13%)	
Endometrial	17	2.30 (0–41.45)		13 (76.47%)	4 (23.53%)	
stage			0.001^c^			0.03
Stage I	21	0 (0–3.20)		21 (100%)	0 (0%)	
Stage II	34	0 (0–4.80)		30 (88.24%)	4 (11.76%)	
Stage III	43	0.83 (0–22.26)		34 (79.07%)	9 (20.93%)	
Stage IV	15	2.92 (0–41.45)		8 (53.33%)	7 (46.67%)	
NA	4	2.62 (0.45–13.80)		3 (75%)	1 (25%)	
HPV status (Cervix cancer)			0.506			0.255
positive	9	0 (0–9)		7 (77.78%)	2 (22.22%)	
negative	5	2 (0–7)		5 (100%)	0 (0%)	
uncertain	18	2.28 (0–40.81)		11 (61.11%)	7 (38.89%)	
Subtype of Endometrial Carcinoma			—			—
Type I	16	2.30 (0–41.45)		12 (75%)	4 (25%)	
Type II	1	0.95		1 (100%)	0 (0%)	
Tissue types			0.34			0.18
Serous adenocarcinoma	60	0.36 (0–22.26)		52 (86.67%)	8 (13.33%)	
Clear cell adenocarcinoma	3	0.38 (0–3.20)		3 (100%)	0 (0%)	
Endometroid adenocarcinoma	17	2.30 (0–41.45)		13 (76.47%)	4 (23.53%)	
Squamous cell carcinoma	31	0.99 (0–40.81)		22 (70.97%)	9 (29.03%)	
Mucinous adenocarcinoma	1	0		1 (100%)	0 (0%)	
Other	5	0 (0–2.30)		5 (100%)	0 (0%)	

Note: ^a^calculated using Mann-Whitney test/Kruskal-Wallis test followed by Dunn’s multiple comparison as post-hoc test. ^b^Calculated using Pearson’s chi-square test. ^c^Calculated using Kruskal-Wallis test followed by Dunn’s multiple comparison as post-hoc test, the Dunn’s multiple comparison showed that the difference between stage I and stage IV, stage II and stage IV, satge I and stage III were statistical significant (P = 0.0007, 0.019, and 0.009 respectively).

### Genomic alterations associated with TMB

**Figure 3:**
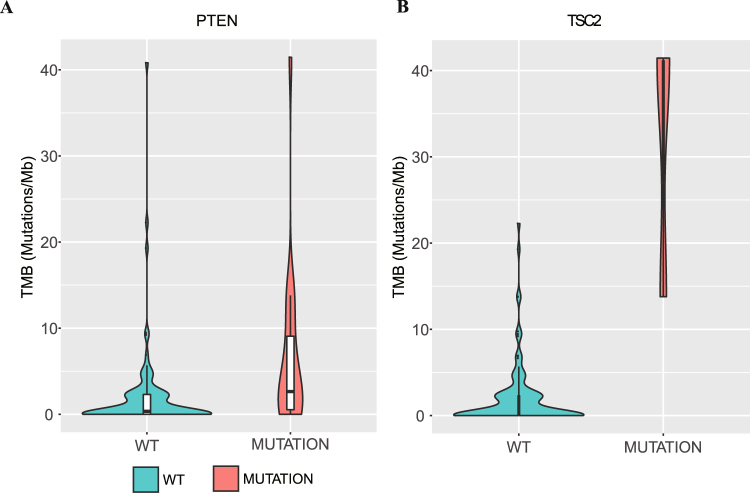
Association of mutations in cancer genes with TMB. (**A**) Plot of mutation burden in specimens with known or likely driver mutations in PTEN (n = 18) and specimens without such mutations (n = 99). (**B**) Plot of mutation burden in specimens with known or likely driver mutations in TSC2 (n = 3) and specimens without such mutations (n = 114).

To explore the association between genomic alterations and TMB, we investigated the molecular profiles of moderate to high TMB across our samples and performed statistical analysis to identify the specific genetic alterations related to increased TMB. Previous studies have shown that *POLE* alterations are associated with TMB in multiple cancer types^28^. In line with previous research, we found that patients with *POLE* mutations had a significantly higher TMB (Table 3, p = 0.005). In addition to *POLE*, other genomic alterations associated with TMB were identified in our study. Indeed, somatic mutations in *PTEN* were commonly found in patients with a moderate to high TMB (n = 8, 38.10%) (Fig. 3A and Table 3). Moreover, limited by the sample size, *TSC2* mutations were detected in only 3 patients (2.56%). However, all three of these patients had a high TMB (Fig. 3B, Table 3 and Supplementary Fig. S1). In addition to *PTEN*, *POLE* and *TSC2*, the gene mutation status of *BRCA1*, *FBXW7*, *PIK3R1* and *STK11* differed significantly according to TMB (Table 3 and Supplementary Fig. S1). Patients with mutated *BRCA1* (4, 57.14%), *FBXW7* (3, 60%), *PIK3R1* (2, 66.67%) and *STK11* (1, 50%) tended to have higher TMB levels. However, due to the limited sample size, these four genes need to be further confirmed in a large study. Although *TP53* alterations are commonly seen in gynecologic cancer, it was observed in only five patients with a moderate to high TMB in our cohort (Table 3). Furthermore, there was no significant difference in TMB between patients with or without *TP53* mutations (p = 0.26).

**Table 3:** The association of TMB levels and genomic alterations.

	mutation status	TMB, median (range), mutations/Mb	P value^a^	TMB level		P value^b^
				Low (%)	Moderate to high (%)	
BRCA1	mutated	5.68 (0–22.26)	0.015	3 (42.86%)	4 (57.14%)	0.005
	wild type	0.36 (0–41.45)		93 (84.55%)	17 (15.45%)	
FBXW7	mutated	4.80 (0.45–22.26)	0.003	2 (40%)	3 (60%)	0.012
	wild type	0.36 (0–41.45)		94 (83.93%)	18 (16.07%)	
PIK3R1	mutated	13.77 (2.30–13.79)	4.46E-05	1 (33.33%)	2 (66.67%)	0.026
	wild type	0.36 (0–41.45)		95 (83.33%)	19 (16.67%)	
POLE	mutated	23.73 (6.66–40.81)	0.005	0 (0%)	2 (100%)	0.002
	wild type	0.36 (0–41.45)		96 (83.48%)	19 (16.52%)	
PTEN	mutated	2.64 (0–41.45)	0.000995	10 (55.56%)	8 (44.44%)	0.001
	wild type	0.35 (0–40.81)		86 (86.87%)	13 (13.13%)	
STK11	mutated	20.41 (0–40.81)	0.028	1 (50%)	1 (50%)	0.234
	wild type	0.37 (0–41.45)		95 (82.61%)	20 (17.39%)	
TSC2	mutated	40.81 (13.78–41.45)	3.83E-11	0 (0%)	3 (100%)	0.005
	wild type	0.36 (0–22.26)		96 (84.21%)	18 (15.79%)	
AKT1	mutated	1.15 (0–2.30)	0.802	2 (100%)	0 (0%)	0.505
	wild type	0.37 (0–41.45)		94 (81.74%)	21 (18.26%)	
ARID1A	mutated	1.15 (0–9.77)	0.135	7 (70%)	3 (30%)	0.299
	wild type	0.37 (0–41.45)		89 (83.18%)	18 (16.82%)	
ATR	mutated	2.84 (0–5.68)	0.245	1 (50%)	1 (50%)	0.234
	wild type	0.37 (0–41.45)		95 (82.61%)	20 (17.39%)	
BRAF	mutated	2.98 (0.69–4.80)	0.488	2 (66.67%)	1 (33.33%)	0.482
	wild type	0.37 (0–41.45)		94 (82.46%)	20 (17.54%)	
BRCA2	mutated	4.25 (0–22.26)	0.166	2 (50%)	2 (50%)	0.089
	wild type	0.36 (0–41.45)		94 (83.19%)	19 (16.81%)	
CTNNB1	mutated	0 (0–22.26)	0.313	4 (80%)	1 (20%)	0.903
	wild type	0.38 (0–41.45)		92 (82.14%)	20 (17.86%)	
FAT1	mutated	2.40 (0–4.80)	0.245	1 (50%)	1 (50%)	0.234
	wild type	0.37 (0–41.45)		95 (82.61%)	20 (17.39%)	
FGFR2	mutated	2.30 (0–6.66)	0.488	2 (66.67%)	1 (33.33%)	0.482
	wild type	0.37 (0–41.45)		94 (82.46%)	20 (17.54%)	
HRAS	mutated	3.2	0.896	1 (100%)	0 (0%)	0.639
	wild type	0.37 (0–41.45)		95 (81.90%)	21 (18.10%)	
KRAS	mutated	0.22 (0–4.53)	0.782	5 (83.33%)	1 (16.67%)	0.933
	wild type	0.37 (0–41.45)		91 (81.98%)	20 (18.02%)	
NF1	mutated	0.37	0.896	1 (100%)	0 (0%)	0.639
	wild type	0.37 (0–41.45)		95 (81.90%)	21 (18.10%)	
PIK3CA	mutated	1.34 (0–40.81)	0.114	12 (66.67%)	6 (33.33%)	0.061
	wild type	0.36 (0–41.45)		84 (84.85%)	15 (15.15%)	
TP53	mutated	0.76 (0–22.26)	0.265	41 (89.13%)	5 (10.87%)	0.108
	wild type	0 (0–41.45)		55 (77.46%)	16 (22.54%)	

Note: ^a^calculated using Mann-Whitney test/Kruskal-Wallis test. ^b^Calculated using Pearson’s chi-square test.

## Discussion

To the best of our knowledge, the present study is the first to assess TMB in Chinese patients with gynecologic cancers and to describe the mutational profiles that are associated with an increased TMB. Tumor mutations are a key mechanism for the generation of anticancer immunity^29^. It is hypothesized that highly mutated tumors are more likely to harbor neoantigens that make them targets of activated immune cells^22–24^. In fact, in one clinical trial, TMB was more significantly associated with RR than was PD-L1 expression by immunohistochemistry^25^. Neoantigen load has also been linked with response to immunotherapy^26^. Whole exome sequencing (WES) has been previously used to measure TMB; however, smaller gene panels have been recently used to measure TMB with equal accuracy as that of WES and to determine the association of TMB with response to immunotherapy^25,26^. In this study, we used a 1.15-Mb panel representing 1086 cancer-related genes to measure TMB in Chinese patients with gynecologic cancers. Both WES and our designed small 1.15-Mb panel were used to calculate the TMB of 25 cancer specimens. The TMB values based on the designed small panel were consistent with those determined by WES (R^2^ = 0.734, data not shown), indicating that the designed small panel is an accurate, cost-effective and clinically available tool for measuring TMB.

Overall the median TMB in gynecologic cancers is 0.37 mutations/Mb, with a range of 0–41.45 mutations/Mb. Most of the patients (82.05%) had a low TMB, while only a few patients (5.98%) had a high TMB. Although high TMB values were observed in all types of gynecologic cancers, endometrial cancer cases had a higher median TMB than ovarian and cervical cancer cases, and 23.53% of patients with endometrial cancer had a moderate to high TMB, indicating that immune checkpoint blockade may be a potential treatment for endometrial cancer. In previous clinical studies, pembrolizumab showed durable anti-tumor activity in a subset of endometrial cancer patients^16,30^. In contrast, the median TMB in ovarian cancer was lower than that in endometrial and cervical cancer. In addition, most of the ovarian cancer patients (88.24%) had a low TMB, and only one patient had a high TMB. These results were consistent with clinical findings that immune checkpoint inhibitors result in limited tumor response in ovarian cancer^19,31^. Previous researchers have reported PD-L1 expression in 95% of cervical intraepithelial neoplasia and 80% of squamous cell carcinomas^11^, and lymph nodes harboring metastatic cervical cancer were characterized by high levels of PD-L1+ antigen-presenting cells (APCs) and FOXP3+ regulatory T (Treg) cells^12^. Although the median TMB in cervical cancer cases was not high, moderate to high TMB values were observed in a subset of patients (28.13%), which may be a potential group that can benefit from immune checkpoint inhibitor therapy. Several ongoing trials are investigating immune checkpoint inhibitors alone or in combination with chemotherapy or other biological agents in patients with advanced and recurrent cervical cancer, but the results have not been published yet^32^.

Understanding the factors associated with genomic instability is also important to better comprehend carcinogenesis and progression. Previous researchers have shown that patients with *POLE* mutations have a significantly higher TMB than patients without *POLE* mutations^28^. We investigated the molecular profiles of patients with a moderate to high TMB and characterized the distribution of somatic mutations in known genes. Similar to previous studies, we observed two patients with *POLE* mutations – one patient had a moderate TMB, and one patient had a high TMB – and TMB values were significantly increased in patients with *POLE* mutations. In addition to *POLE*, we identified several other genes associated with high TMB. Alterations in *TSC2* were associated with a large increase in TMB, although we identified only three cases with single nucleotide variants (SNVs) in this gene. There was another gene, *PTEN*, that was significantly associated with TMB. *PTEN* mutations were commonly seen in patients with a moderate to high TMB. Both *PTEN* and *TSC2* are tumor suppressor genes involved in the PI3K/MTOR/AKT pathway, which is commonly activated in gynecologic cancers^33–35^. According to previous studies, PTEN is correlated with PD-1/PD-L1 expression in lung cancer, and it also plays a role in maintaining genomic integrity during processes such as DNA replication and chromosome segregation^36^. In addition to *PTEN*, *POLE*, and *TSC2*, the gene mutation status of *BRCA1*, *FBXW7*, *PIK3R1*, and *STK11* was related to TMB level. Both *FBXW7* and *PIK3R1* are involved in the PI3K/MTOR/AKT pathway^37,38^. The relationship among *FBXW7*, *PIK3R1*, and immune check point inhibitors remains to be elucidated. *BRCA1*, a tumor suppressor gene, plays an important role in gynecologic cancers, especially ovarian cancer^39^. In 2016, Strickland *et al*. reported that high grade serous ovarian cancers with BRCA1/2 mutations and a high number of tumor-infiltrating lymphocytes (TILs) were associated with elevated expression of PD-1/PD-L1^40^. Loss of STK11 or STK11 somatic mutations may affect the progression of gynecologic cancers^41,42^. According to previous studies, STK11 deficiency in patients with lung cancer is associated with decreased expression of PD-1/PD-L1 and a reduced response rate to immune check point inhibitors^43,44^. Due to the limited sample size, the associations of the *BRCA1*, *FBXW7*, *PIK3R1*, and *STK11* genes with TMB must be further verified in a larger cohort.

In conclusion, we investigated the landscape of TMB in Chinese patients with gynecologic cancers. Patients with mutations in the *PTEN*, *TSC2* or *POLE* gene have an increased TMB. Further large-scale, prospective studies are needed to validate our findings.

## Materials and Methods

### Patient cohort

Formalin-fixed paraffin-embedded (FFPE) tumor specimens were collected at the Department of Gynecology at Chinese People’s Liberation Army (PLA) General Hospital. Clinical data regarding age at first diagnosis, diagnosis, and histology were collected from patient records. The clinical characteristics of the patients are listed in Table 1. The cohort consisted of 64 patients with ovarian cancer, 1 patient with primary peritoneal cancer, 3 patients with fallopian tube cancer, 32 patients with cervical cancer, and 17 patients with endometrial cancer.

### Ethics statement

The study protocol was approved by the Committee of Medical Ethics of the Chinese PLA General Hospital and carried out according to the principles of the Declaration of Helsinki. All patients provided written informed consent to participate in the study prior to their enrollment. All experiments were carried out in accordance with relevant guidelines and regulations.

### DNA extraction, library construction and next-generation sequencing (NGS) analysis

FFPE tissue specimens and matched blood samples were collected from 117 patients who were diagnosed with gynecologic cancer. Tumor DNA was isolated from FFPE specimens with the blackPREP FFPE DNA Kit (Analytik Jena AG, Jena, Germany) according to the manufacturer’s instructions. Each DNA sample was obtained from four 2-μm tissue specimens and quantified with the Qubit dsDNA HS Assay kit (Life Technologies, USA) according to the manufacturer’s recommended protocol. Blood lymphocytes were isolated by centrifugation of whole blood at 1600 g for 10 min at room temperature. Tiangen whole blood DNA kits (Tiangen, Beijing, PRC) were used to extract DNA from peripheral blood lymphocytes according to the manufacturer’s instruction. Tumor DNA and matched genomic DNA were sheared into 150–200-bp fragments by a Covaris M220 Focused-Ultrasonicator (Covaris, Massachusetts, USA). Fragmented DNA libraries were constructed with a KAPA HTP Library Preparation Kit (Illumina Platform) (KAPA Biosystems, Massachusetts, USA) according the manufacturer’s instruction. DNA libraries were captured with a designed 1086-gene panel, NimbleGen SeqCap EZ Library (Roche, Wisconsin, USA), that includes major tumor-related genes. The captured samples were then subjected to Illumina HiSeq X-Ten for paired-end sequencing.

### Identification of somatic mutations and assessment of tumor mutational burden (TMB)

FFPE tumor samples and blood lymphocytes samples were submitted for NGS. The NimbleDesign assay was used (1086 genes) to identify mutations. We used VarScan2 with the following filters: (i) located in intergenic regions or intronic regions; (ii) synonymous SNVs; (iii) allele frequency >= 0.002 in the database exac03 or gnomad_exome; (iv) the value of ljb2_pp2hdiv = “B” and ljb2_pp2hvar = “B”; (v) allele frequency <0.05 in the tumor sample; and (vi) allele depth <5. To identify somatic mutations, the mutations in FFPE tumor samples were blanked by matched blood lymphocyte samples from patients. For the determination of TMB, the number of somatic nonsynonymous SNVs (with depth >100X and allele frequency ≥ 0.05) detected on NGS (interrogating Mb of the genome) were quantified, and the value was extrapolated to the whole exome using a validated algorithm. Alterations likely or known to be bona fide oncogenic drivers were excluded. TMB was measured in mutations per Mb. TMB values were divided into the following three groups: low (<3.24 mutations/Mb), intermediate (3.24–12.94 mutations/Mb), and high (≥12.94 mutations/Mb).

### Statistical methods

Both the Mann-Whitney test/Kruskal-Wallis test followed by Dunn’s multiple comparison as post-hoc test and Pearson’s chi-square test were used to analyze the significance of the association of TMB with disease site, patient age and tumor type. The significance of associations of the number of gene mutations with TMB and disease site was also analyzed with Kruskal-Wallis tests. All tests were two-sided, and statistical significance was set at p < 0.05. All statistical analyses were performed with SPSS version 22.0 software (SPSS Inc., Chicago, IL, USA).

### Data avaliability statement

The datasets generated and analyzed during the current study are available from the corresponding author on reasonable request.

## Electronic supplementary material

Supplemental Figure S1
