# Supplementary material for: Molecular profiles and tumor mutational burden analysis in Chinese patients with gynecologic cancers
Source: Sci Rep. 2018 Jun 12;8:8990. doi: 10.1038/s41598-018-25583-6 (PMC5997642; doi:10.1038/s41598-018-25583-6)
Supplement: Supplementary file 1 — Supplemental Figure S1 [file 41598_2018_25583_MOESM1_ESM.pdf]

Molecular profiles and tumor mutational burden analysis in Chinese patients with gynecologic cancers

Min Wang, Wensheng Fan, Mingxia Ye, Chen Tian, Lili Zhao, Jianfei Wang, Wenbo Han, Wen Yang, Chenglei Gu, Mingxia Li, Zhe Zhang, Yongjun Wang, Henghui Zhang, Yuanguang Meng.

Supplementary Table S1. Cancer related gene list.

A

ABALON	ABCA1	ABCA3	ABCB1	ABCB4	ABCB6	ABCB11
ABCC1	ABCC2	ABCC3	ABCC4	ABCC5	ABCC6	ABCC9
ABCC11	ABCG1	ABCG2	ABHD18	ABL1	ACE	ACHE
ACO1	ACP5	ACPP	ACSM2A	ACSS2	ACTA2	ACTB
ACTG1	ACTR8	ACVR2B	ACYP2	ADAM22	ADAMTSL4-AS1	ADCY2
ADCY10	ADD1	ADGRB3	ADH1A	ADH1B	ADH1C	ADH7
ADIPOQ	ADIPOQ-AS1	ADRA2B	ADRA2C	ADRB1	ADRB2	ADRB3
AFMID	AGO1	AGO2	AGT	AGTR1	AGTRAP	AGXT
AHR	AKAP9	AKNA	AKR1C2	AKR1C3	AKT1	AKT2
ALB	ALDH1A1	ALDH3A1	ALDH5A1	ALK	ALOX12	ALOX12-AS1
ANK1	ANKK1	ANTXR2	ANXA11	AOC1	AOX1	APC
APCS	APEX1	APOA1	APOA1-AS	APOA4	APOA5	APOB
APOBEC2	APOC3	APOE	APOH	AQP2	AQP9	AR
ARAF	AREG	ARHGEF4	ARHGEF10	ARID1A	ARID5B	ARNT
ARRDC3	ART3	ASH1L	ASH1L-AS1	ASS1	ATG5	ATIC
ATM	ATP7A	ATP7B	ATR	ATXN2L	AURKA	AXIN2
AZGP1	AZU1					

B

B2M	BAIAP2L1	BAP1	BAZ2B	BCAT1	BCHE	BCL2
BCL2L1	BCL2L11	BCOR	BCR	BDNF	BDNF-AS	BGLAP
BIVM-ERCC5	BLMH	BMP7	BMX	BRAF	BRCA1	BRCA2
BRD2	BRD4	BRWD1	BTK	BTN3A1		

C

C2	C5ORF22	C8ORF34	C10ORF11	C10ORF55	C11ORF65	C20ORF194
CA2	CA4	CA12	CA13	CA14	CACNA2D3	CALM3
CAMK1	CAMK2A	CAMK4	CANT1	CAPG	CARD16	CARS
CARTPT	CASC8	CASP1	CASP3	CASP7	CASP9	CAT
CBL	CBR1	CBR3	CBR3-AS1	CBS	CBSL	CCAT2
CCDC6	CCDC159	CCL1	CCND1	CCND2	CCND3	CCNE1
CCNH	CCNY	CD1D	CD3EAP	CD14	CD28	CD40LG
CD47	CD55	CD74	CD180	CD274	CDA	CDC20B
CDH1	CDK2	CDK4	CDK6	CDK7	CDK8	CDKN1A
CDKN1B	CDKN2A	CDKN2B	CDKN2B-AS1	CDKN2C	CEACAM22P	CEP89
CES2	CFTR	CHAT	CHEK2	CHRNA3	CHRNA5	CHST3
CHURC1-FNTB	CLCN6	CLDN34	CLIC1	CLIP1	CLK3	CLTC
CMPK1	CMTR1	CNOT1	CNR1	COL1A1	COL1A2	COL4A1
COLEC10	COMT	CPA1	CPA2	CPA4	CPB2	CPB2-AS1
CRABP2	CRAT	CREBBP	CRP	CRYZ	CSDE1	CSF1R
CSF2RB	CSF3R	CSMD3	CSNK2A1	CSPG4	CTLA4	CTNNB1
CTSA	CTSL	CTSS	CXCL8	CXCL12	CXCR2	CXXC4
CYBA	CYP1A1	CYP1A2	CYP1B1	CYP2A6	CYP2B6	CYP2C8

CYP2C19	CYP2D6	CYP2E1	CYP2R1	CYP3A4	CYP3A5	CYP4B1
CYP4F2	CYP19A1	CYP24A1	CYP46A1			

D

DAPK1	DAZL	DCBLD1	DCK	DCP1B	DCTD	DCTN1
DDR1	DDR2	DDX6	DDX53	DEAR	DERL3	DGKI
DHFR	DLG4	DLG5	DLGAP1	DNMT3A	DOCK4	DOT1L
DPEP1	DPP4	DPYD	DPYD-AS1	DPYS	DRD2	DROSHA
DSCAM	DTYMK					

E

E2F7	ECT2L	EFNB2	EGF	EGFR	EGFR-AS1	EGLN3
EIF3A	EIF4E2	EML4	ENOSF1	EPAS1	EPHA2	EPHA3
EPHA4	EPHA5	EPHA6	EPHX1	EPRS	ERAP2	ERBB2
ERBB3	ERBB4	ERC1	ERCC1	ERCC2	ERCC3	ERCC4
ERCC5	ERCC6	EREG	ERG	ERP44	ESR1	ESR2
ESRP1	ETF1	ETFRF1	ETV1	ETV4	ETV5	ETV6
EVI2A	EXO1	EZH2	EZR			

F

F2	F10	FAAH	FABP1	FAM131B	FAM212B	FANCA
FANCC	FAP	FARS2	FAT1	FBXW7	FCGR2A	FCGR3A
FCHSD1	FCN1	FCN2	FDPS	FECH	FGD4	FGF3
FGF4	FGFR1	FGFR2	FGFR3	FGFR4	FIBCD1	FKBP4
FKBP5	FLCN	FLT1	FLT3	FLT4	FMO2	FMO3
FN1	FNTA	FOLH1	FOLR2	FOLR3	FOXA1	FRS2
FSTL5	FTO	FZD3				

G

GABBR1	GABBR2	GAK	GALE	GALNS	GALNT10	GALNT14
GALNT18	GAPDH	GARS	GATA3	GATA4	GATM	GCKR
GDNF	GEMIN4	GGCT	GGH	GIPR	GLP1R	GLRB
GLRX	GLRX2	GNA11	GNAI1	GNAQ	GNAS	GNAS-AS1
GNB3	GNL3	GOLGA5	GOPC	GP1BA	GPC1	GPX5
GRIK1	GRIN2B	GRK5	GSG2	GSR	GSS	GSTA1
GSTA2	GSTA3	GSTA5	GSTM3	GSTO1	GSTP1	GSTT1
GUSB						

H

H19	HACL1	HADH	HAS3	HAT1	HCN4	HDAC1
HDAC2	HDAC8	HERPUD1	HEXB	HFE	HGF	HIF1A
HIP1	HLA-A	HLA-B	HLA-C	HLA-DOA	HLA-DOB	HLA-DPA1
HLA-DPB1	HLA-DQA1	HLA-DQB1	HLA-DRA	HLA-DRB1	HLA-G	HMGB1

HMGCR	HNF4A	HNMT	HNRNPUL1	HOOK1	HOOK3	HOTAIR
HOTTIP	HPGDS	HRAS	HSCB	HSP90AA1	HSPA5	HSPA8
HTR2A	HTR2C	HTR3B	HYKK			

I

IDH1	IDH1-AS1	IDH2	IFNG	IFNL3	IFNL4	IGF1
IGF1R	IGF2	IGF2BP2	IGFBP3	IL1B	IL1RL1	IL6R
IL6ST	IL7R	IL13	IL17F	IL20RA	IL21R-AS1	IL23R
IMPDH1	INPP4B	INS-IGF2	IRS1	ITGA2B	ITGA4	ITGA5
ITGAE	ITGAV	ITGAX	ITGB1	ITGB2	ITGB2-AS1	ITGB5
ITPA						

J

JAG1	JAK1	JAK2	JAK3	JMJD6

K

KARS	KCNMB3	KCNQ1	KCNQ5	KDM4D	KDR	KEAP1
KIF5B	KIT	KLC1	KLC3	KLHL12	KLLN	KMT5A
KMT5B	KRAS	KRT19P2	KSR2	KYAT1		

L

LAMP3	LANCL1	LANCL1-AS1	LARP1B	LDHA	LDHB	LDLR
LEP	LEPR	LGALS3BP	LGR5	LIG3	LIG4	LIN7A
LINC00673	LINC00704	LIPF	LMNA	LOC286059	LOC541472	LOC553103
LOC100128714	LOC100133286	LOC100507053	LOC100507291	LOC100507346	LOC100996325	LOC101927066
LOC101927318	LOC101927768	LOC101928100	LOC101928402	LOC101928414	LOC101928718	LOC101929532
LOC101929829	LOC102724050	LPL	LRIG3	LRP1B	LRP2	LRP5
LRP6	LRRC4C	LRTM1	LSM14A	LTA4H	LY86	LY96

M

MAD1L1	MALAT1	MALT1	MAOB	MAP2K1	MAP2K2	MAP3K1
MAP3K5	MAP3K7	MAPK11	MAPK14	MCL1	MDM2	MDM4
MECP2	MEG3	MEP1B	MET	METAP1	MFSD11	MGAM
MGAT4A	MGMT	MICB	MIEN1	MIR27A	MIR146A	MIR300
MIR323B	MIR423	MIR449B	MIR492	MIR577	MIR604	MIR618
MIR636	MIR1206	MIR1307	MIR2053	MIR3142HG	MIR3184	MIR4673
MIR4728	MIR4761	MIR6759	MIR6891	MISP	MITD1	MITF
MKRN1	MLH1	MLLT3	MMAB	MME	MMP8	MMP13
MPL	MRM2	MSH2	MSH3	MSN	MST1R	MTAP
MTHFD1	MTHFR	MTOR	MTR	MTRR	MUTYH	MVK

MVP	MYC	MYD88	MYLIP	MYO5A

N

NAGA	NALCN	NAMPT	NAT2	NCAM2	NCOA1	NCOA4
NEDD4	NEU2	NF1	NF2	NFASC	NFATC2	NLGN1
NLGN4X	NMRAL1	NOD2	NOS3	NOTCH1	NOTCH2	NPAT
NPC1	NPM1	NQO1	NQO2	NR1I2	NR1I3	NR3C1
NR3C2	NRAS	NRG1	NSD1	NSRP1	NT5C2	NT5C3A
NT5E	NTHL1	NTRK1	NTRK3	NUDC	NUDT2	NUDT15
NUP210L						

O

OASL	OGG1	OPRD1	OPRM1	OR4D6	OTOS

P

PACERR	PACSIN2	PAGR1	PAH	PAK1	PALB2	PAPD7
PARD3B	PARPBP	PAX5	PBRM1	PC	PCK1	PCM1
PDCD1	PDE4B	PDE5A	PDE10A	PDGFB	PDGFRA	PDGFRB
PDK3	PDPK1	PDXK	PDZRN3	PFKFB3	PGAP3	PGBD3
PGR	PHF8	PHKG2	PIGF	PIK3CA	PIK3CB	PIK3R1
PIK3R2	PIM1	PINLYP	PKD1	PLA2G7	PLAU	PLCB1
PLCB3	PLCG2	PLG	PLXNC1	PMF1-BGLAP	PMM2	PNMT
PNP	POFUT2	POLA2	POLE	POLK	POLR2F	PON1
POR	POU3F2	PPARA	PPARD	PPARG	PPFIBP1	PPIF
PPIP5K2	PPP1R13L	PRB2	PRCP	PRDX4	PREP	PRKAA2
PRKAR1A	PRKCH	PRKCI	PRKG1	PROM1	PSMA8	PTCH1
PTCHD1-AS	PTEN	PTGES	PTGR1	PTGS2	PTH	PTH1R
PTK2	PTPN1	PTPN23	PTPRG	PTPRK	PTPRM	PTRH2
PUS1	PVT1	PWWP2A	PYGL	PYGM		

R

RAB3D	RAC1	RAC2	RAD50	RAD51C	RAD52	RAF1
RALBP1	RANBP2	RAPGEF5	RARB	RARG	RARS	RAVER2
RB1	RBFOX1	RBP2	RBP4	REN	RET	REV1
REV3L	RFK	RGS5	RHOQ	RICTOR	RIPK2	RNASE2
RNF43	RNF128	RNF130	RNU5F-1	RORC	ROS1	RRAS2
RRM1	RRM2	RUNDC3B	RXRA			

S

SCARB1	SCN1A	SCN10A	SCO2	SDC4	SDCBP	SEC16A
SEC31A	SELE	SELL	SEMA3C	SERPINA3	SERPINA6	SERPINA7
SERPINE1	SERPING1	SETD2	SETD3	SETD6	SF3B1	SFXN4
SH2B3	SH2D5	SHMT1	SHTN1	SIGLEC7	SIRPA	SIRT2

SLC1A1	SLC2A1-AS1	SLC3A2	SLC6A4	SLC7A5	SLC10A2	SLC15A1
SLC19A1	SLC22A1	SLC22A2	SLC22A4	SLC22A5	SLC22A7	SLC22A12
SLC22A16	SLC25A13	SLC28A1	SLC28A2	SLC28A3	SLC29A1	SLC31A1
SLC34A2	SLC37A1	SLC45A3	SLCO1A2	SLCO1B1	SLCO1B3	SLCO4C1
SMARCA1	SMARCA4	SMARCAD1	SMARCB1	SMO	SMS	SMYD2
SMYD3	SNAPC5	SND1	SNRNP200	SOCS1	SOD2	SOD3
SOX10	SPARC	SPG7	SPIDR	SPON1	SQSTM1	SRC
SRGAP3	SRSF2	SRXN1	SSBP2	ST14	STAG2	STIM1
STK11	STRADA	STRN	STS	SULT1A1	SULT1C4	SULT1E1
SULT2B1	SUV39H2	SUZ12	SVIL	SVOP	SYK	SYN1
SYNE1						

T

TAB1	TACC3	TANC1	TAS2R16	TBC1D1	TBC1D20	TCN1
TDP1	TEK	TENM1	TF	TFG	TGFB1	TGM2
TH2LCRR	THRA	THRB	TIMM23B	TKT	TLR2	TLR3
TLR4	TLR7	TLR9	TMPRSS2	TNFRSF10B	TNFRSF11A	TNFRSF11B
TNFSF11	TNFSF14	TNIP1	TNNC1	TNRC6A	TNRC6B	TOMM40L
TOP1	TOP2A	TOP2B	TP53	TPBG	TPH2	TPM3
TPM4	TPMT	TPP1	TRIM24	TRIM27	TRIM33	TRMT61B
TRPV4	TSC1	TSC2	TSG1	TSHR	TSPAN31	TSPOAP1-AS1
TTC6	TTK	TTR	TUBB1	TUBB2A	TXNRD2	TYMP
TYMS	TYMSOS	TYW3				

U

U2AF1	U2AF1L5	UBASH3B	UBC	UBE2D1	UBE2I	UGT1A1
UGT1A3	UGT1A4	UGT1A5	UGT1A6	UGT1A7	UGT1A8	UGT1A9
UGT1A10	UGT2B7	UGT8	ULBP3	ULK3	ULK4	UMPS
UPB1	UPP1	USMG5	USP6	UTY		

V

VAC14	VCL	VCPKMT	VDR	VEGFA	VEGFC	VHL
VKORC1	VWF					

W

WAPL	WARS	WDR7	WRAP53	WWOX

X

XDH	XPA	XPC	XPO5	XRCC1	XRCC3	XRCC4
XRCC5						

Z						
ZADH2	ZBTB17	ZCCHC6	ZNF276	ZNF397	ZNF423	ZNRF3
ZPR1	ZSCAN30					

		TMB, median (range), mutations/Mb	P value	TMB level		P value
				Low (%)	Moderate to high (%)	
Age, median (range), years			0.37			0.61
<40	14	0 (0-4.80)		13 (92.86%)	1 (7.14%)	
40-49	26	2.01 (0-13.7965)		20 (76.92%)	6 (23.08%)	
50-59	43	0.37 (0-41.45)		36 (83.72%)	7 (16.28%)	
>=60	34	0.52 (0-22.26)		27 (79.41%)	7 (20.59%)	
Tissue types (in each cancer types)						
Ovarian cancer			-			-
Serous adenocarcinoma	59	0.36 (0-22.26)		51 (86.44%)	8 (13.56%)	
Clear cell adenocarcinoma	3	0.38 (0-3.20)		3 (100%)	0 (0%)	
Endometroid adenocarcinoma	1	0		1 (100%)	0 (0%)	
Other	5	0 (0-2.30)		5 (100%)	0 (0%)	
Cervical cancer			-			-
Squamous cell carcinoma	31	0.99 (0-40.81)		22 (70.97%)	9 (29.03%)	
Mucinous adenocarcinoma	1	0		1 (100%)	0 (0%)	
Endometrial cancer			-			-
Endometroid adenocarcinoma	16	2.30 (0-41.45)		12 (75%)	4 (25%)	
Serous adenocarcinoma	1	0.95		1 (100%)	0 (0%)	

Supplemental Table S2. The association of TMB and clinical status.

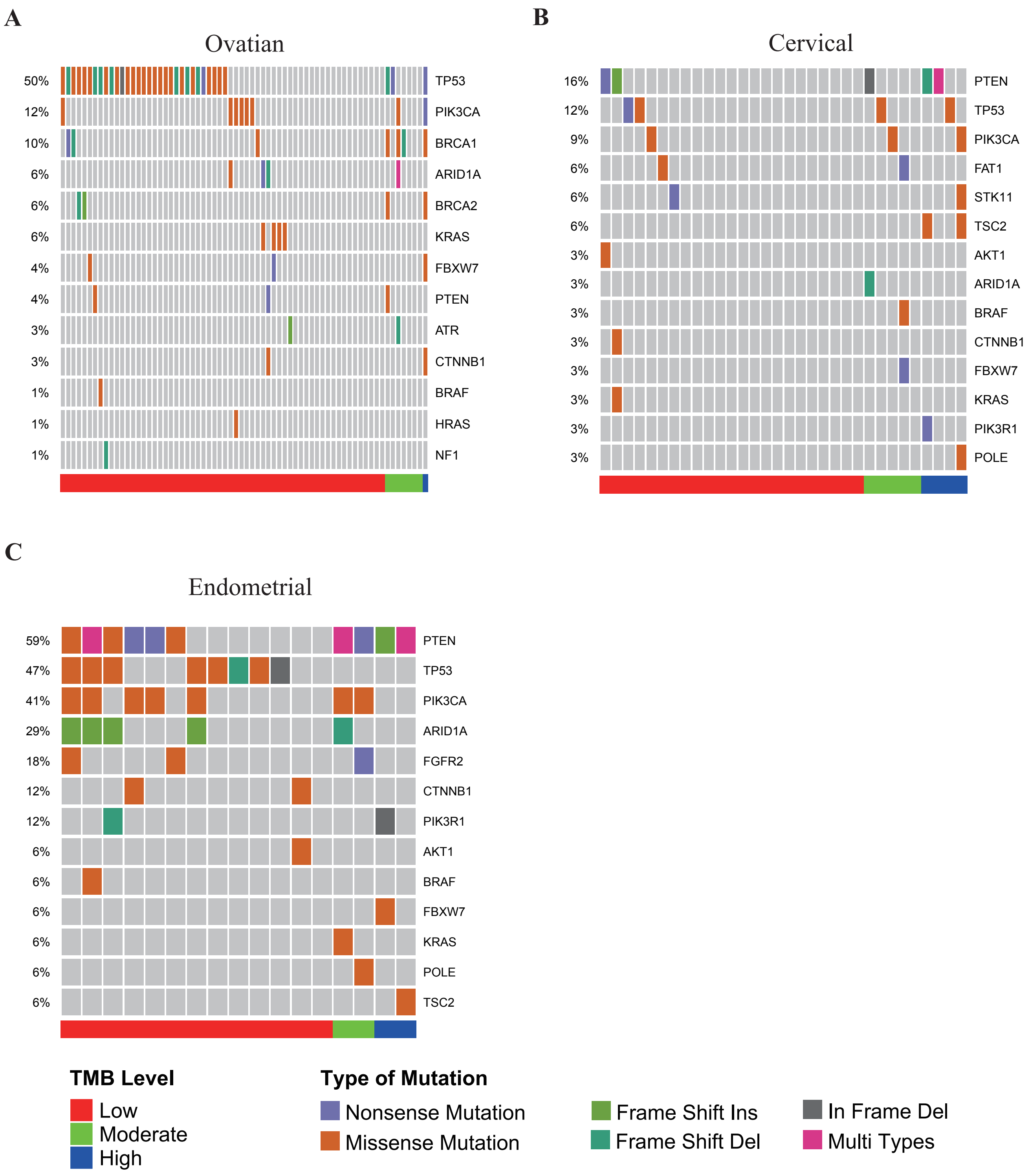

Supplemental Figure S1. Association of mutations in cancer genes with TMB in each cancer type. Association of mutations in cancer genes with TMB in ovarian cancer (a), cervical cancer (b), and endometrial cancer (c).
